# Supplementary material for: Splice-Junction-Based Mapping of Alternative Isoforms in the Human Proteome
Source: Cell Rep. Author manuscript; Available in PMC 2020 Jan 15. (PMC6961840; doi:10.1016/j.celrep.2019.11.026)
Supplement: 2 [file NIHMS1546469-supplement-2.zip › DF1/PXD010154/Liver/Liver_21_TRIM28_RSGAAHSAMCSLTAAAASAASGSPGPGEGSAGGEK.pdf]

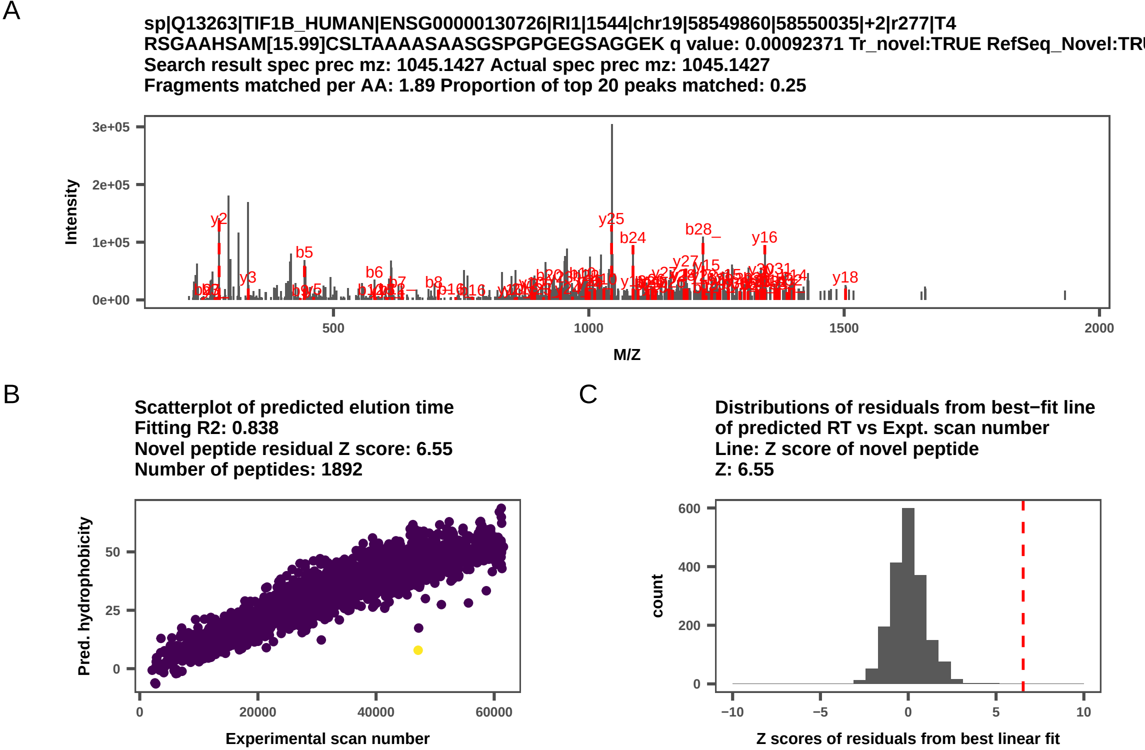
A
sp|Q13263|TIF1B_HUMAN|ENSG00000130726|RI1|1544|chr19|58549860|58550035|+2|r277|T4
RSGAAHSAM[15.99]CSLTAAAASAASGSPGPGEGSAGGEK q value: 0.00092371 Tr_novel:TRUE RefSeq_Novel:TRU
Search result spec prec mz: 1045.1427 Actual spec prec mz: 1045.1427
Fragments matched per AA: 1.89 Proportion of top 20 peaks matched: 0.25
Intensity
3e+05
2e+05
1e+05
0e+00
y2
y3
b5
b6
b8
y25
b24
b28_
y27
y15
y16
y18
500
1000
1500
2000
M/Z
B
Scatterplot of predicted elution time
Fitting R2: 0.838
Novel peptide residual Z score: 6.55
Number of peptides: 1892
Pred. hydrophobicity
50
25
0
0
20000
40000
60000
Experimental scan number
C
Distributions of residuals from best−fit line
of predicted RT vs Expt. scan number
Line: Z score of novel peptide
Z: 6.55
count
600
400
200
0
−10
−5
0
5
10
Z scores of residuals from best linear fit
